# Antioxidant Response of Chronic Wounds to Hyperbaric Oxygen Therapy

**DOI:** 10.1371/journal.pone.0163371

**Published:** 2016-09-21

**Authors:** Antoni Sureda, Juan M. Batle, Miquel Martorell, Xavier Capó, Silvia Tejada, Josep A. Tur, Antoni Pons

**Affiliations:** 1 Research Group on Community Nutrition and Oxidative Stress, University of Balearic Islands, and CIBEROBN (Physiopathology of Obesity and Nutrition), E-07122, Palma de Mallorca, Balearic Islands, Spain; 2 Departamento de Nutrición y Dietética, Facultad de Farmacia, Universidad de Concepción, E-4070386, Concepción, Chile; 3 Experimental Laboratory, Research Unit, Son Llàtzer Hospital, IUNICS, Ctra. Manacor km 4, E-07198, Palma de Mallorca, Balearic Islands, Spain; University of Arkansas for Medical Sciences College of Pharmacy, UNITED STATES

## Abstract

We analyzed the effects of the clinical hyperbaric oxygen therapy (HBOT) on the plasma antioxidant response and levels of endothelin-1, Interleukine-6 (IL-6) and vascular endothelial growth factor (VEGF) in patients with chronic wounds (20.2±10.0 months without healing). They received 20 HBOT sessions (five sessions/week), and blood samples were obtained at sessions 1, 5 and 20 before and 2 hours after the HBOT. An additional blood sample was collected 1 month after wound recovery. Serum creatine kinase activity decreased progressively in accordance with the wound healing. Plasma catalase activity significantly increased after the first and fifth sessions of HBOT. Plasma myeloperoxidase activity reported significantly lower values after sessions. Plasma VEGF and IL-6 increased after sessions. Endothelin-1 levels were progressively decreasing during the HBOT, being significant at the session 20. Plasma malondialdehyde concentration was significantly reduced at the last session. Both creatine kinase activity and malondialdehyde levels were maintained lower 1 month after wound recovery respect to initial values. In conclusion, HBOT enhanced the plasma antioxidant defenses and may contribute to activate the healing resolution, angiogenesis and vascular tone regulation by increasing the VEGF and IL-6 release and the endothelin-1 decrease, which may be significant factors in stimulating wound healing.

## Introduction

Hyperbaric oxygen therapy (HBOT) is the clinical utilization of the oxygen at pressures higher than atmospheric pressure, habitually at 2–3 atmosphere absolute (ATA) pressure with 100% oxygen exposure. Breathing oxygen at elevated pressure increases the oxygen availability for the body tissues. Moreover, HBOT increases the capacity of blood plasma to transport oxygen with respect to normobaric conditions. HBOT has been successfully employed to manage diverse clinical diseases [1], such as non-healing diabetic and selected problem wounds, and necrotizing soft tissue infection. The wound healing is a complex process and requires an orderly and coordinated sequence of steps involving the production and participation of many growth factors, components of the extracellular matrix, and several cell types. Tissue repair involves the mobilization and activation of the immune system that is responsible for the clearance of the damaged tissue from dead cells and matrix debris, and for the mediators synthesis that stimulates angiogenesis and fibroblast growth [2]. The repair process includes an inflammatory phase, but also a resolution phase of the inflammation and wound healing returning to tissue homeostasis [2, 3].

The main basis behind the use of HBOT in the management of chronic non-healing wounds is the increase in the amount of oxygen in blood and the generation of a favourable gradient for the diffusion of oxygen into the affected tissues. In most of the pathological situations with delayed wound healing, there is a direct association with the harmful effects of prolonged oxygen deficit [4]. In this way, diverse processes essential for normal wound healing such as fibroblast proliferation, angiogenesis, collagen deposition or resistance to infection are oxygen-dependent [5]. The increase of oxygen levels in the hypoxic wound is essential for the cells involved in the healing process (neutrophils, fibroblasts, macrophages) to carry out their specific repair functions [6]. Linked to this observation, HBOT has been evidenced to diminish the number of major lower limb amputations among diabetic people [7, 8]. The increased oxygen availability during HBOT is an essential mediating factor associated to wound collagen deposition, cross-linking and neovascularization [9]. HBOT is not able to increase the quantity of oxygen bound to hemoglobin molecules, but can raise the quantity of dissolved oxygen in the plasma [10]. Moreover, the increased oxygen availability increases ROS generation which is directly associated with the modulation of the inflammatory process [11–13]. HBOT increases the expression of diverse growth factors and activates the hypoxia-inducible factor 1 (HIF-1) that may enhance angiogenesis and fibroblast proliferation [14, 15]. In addition, most authors have indicated that the resulting hyperoxia may cause vasoconstriction, thereby decreasing tissue oedema [8, 16], although this oedema decrease might be cause by an inflammation reduction after HBOT [17]. HBOT treatment also induces changes in the oxidative capabilities of immune cells, which can actively participate in the process of wound healing [18]. Further significant effects of HBOT associated to wound healing are an enhanced capacity to kill bacteria by leukocytes, suppression of bacterial proliferation due to its bactericidal effect on anaerobes and microaerophilic aerobes, down-regulation of inflammatory pathways by reducing the expression of pro-inflammatory cytokines, and prevention of leukocyte activation after an ischemic reperfusion [11, 19, 20].

HBOT exposure can increase the production of reactive oxygen species (ROS), directly related to the amount of oxygen present. ROS overproduction is detrimental for cells because it can damage cell components including proteins, lipids, and nucleic acids leading to significant alteration of health status [21]. However, a moderate increase in ROS can be beneficial because ROS may also act as cellular messengers in many signal transduction pathways [22]. Accordingly, vascular endothelial growth factor (VEGF) is up-regulated when cells are exposed to both hypoxic and hyperoxic conditions, whereas endothelin-1 seems to be decreased after a depth dive which is related to the hyperoxia condition [23]. VEGF is considered a key promoter of the angiogenic process which is essential for the maintenance of the integrity of blood vessels, while endothelin-1 participates in the maintenance of the basal vascular tone and blood pressure by activating the vascular smooth muscle. In addition, endothelin-1 together with other proteins operate in a network that promotes myofibroblast differentiation and persistence during wound repair [24]. Acute production of IL-6 has direct anti-inflammatory effects or stimulates immune cell production of anti-inflammatory components, such as the IL-1-receptor antagonist and IL-10 [25]. IL-6 treatment of wound enhances healing by switching immune cells to resolution of inflammation [26]. In addition, IL-6 has been reported to enhance mesenchymal stem cells proliferation in order to protect from apoptosis and to increase the rate of *in vitro* wound healing [27].

The aim of this study was to define the time course of various changes in response to HBOT in chronic wounded patients in which conventional treatments have been reported to be ineffective. Specifically, the activities of antioxidant enzymes were measured in erythrocytes and plasma as well as plasma myeloperoxidase activity and malondialdehyde adducts. Moreover, plasma levels of endothelin-1, VEGF and IL-6 were determined.

## Materials and Methods

### Patient characteristics

14 patients, ten men and four women, (65.8 ± 5.8 years old) presenting a chronic non-healing wound situation were recruited and volunteered to participate in the study. The protocol procedures were designed in compliance with the recommendations for clinical research of the Declaration of Helsinki and were revised and approved by the Ethical Committee of Clinical Investigation of the Government of Balearic Islands (Palma de Mallorca, Balearic Islands, Spain) with number IB1295/09PI. At the beginning of the study, 18 patients were recruited but two decided to abandon before the finishing all sessions because they suffered from claustrophobia and another two were excluded due to health complications: one reported ear pain during the first HBOT and the second one suffered a bowel obstruction secondary to radiation enteritis. All the participants were informed about the research protocol before giving their written consent to participate in the study. Seven of the subjects suffered from diabetic foot ulcer, four of them from osteomyelitis, two from enteritis radica and one from osteoarthritis. Previous conventional treatments, included topical antibiotics (selected after bacterial culture and antibiotic sensitivity tests), topical dressings, and debridement of tissue, reported to be ineffective in this kind of patients. In the case of osteomyelitis, which is an infectious process, antibiotics were supplied systemically and topically. All participants were non-smokers and did not take any antioxidant dietary supplement for 1 month before the study. Before beginning the HBOT, all participants passed a standard medical and physical revision at the hospital. During the HBOT, wounds were cleaned with saline solution, treated with antibiotics, and daily wound curettage or debridement of necrotic tissue was performed to get a well-bleeding granulating base.

### Experimental procedure

The protocol procedure consisted of 20 HBOT exposures in a hyperbaric chamber during a month (five sessions/week form Monday to Friday). Patients breathed 100% oxygen at a pressure of 2.2 ATA in the hyperbaric chamber during 1 h.

Blood samples were obtained before and 2 hours after the first, the fifth and the twentieth HBOT sessions. In addition, another blood sample was collected 1 month after ending HBOT with wound recovery and was utilized as control sample for routine analysis plus plasma malondialdehyde (MDA) levels, as an indicator of lipid peroxidation. Venous blood samples were collected from the antecubital vein of patients in appropriate vacutainers. Hematological parameters including blood cell counts, hematocrit and hemoglobin concentration were determined using an automatic flow cytometer analyzer Technicon H2 (Bayer) VCS system following the standard clinical procedures. Creatine kinase (CK) was measured in serum by standard procedures using an autoanalyser (Technicon DAX System).

Plasma was obtained after centrifugation of the blood samples at 900g at 4°C for 30 min. The plasma phase was removed, and the erythrocytes at the bottom were washed with phosphate buffered saline and centrifuged again at 900g for 30 min. Erythrocytes were lysed with distilled water and reconstituted in the same volume as the initial plasma volume. Erythrocytes and plasma were immediately stored at -80°C until use. Biochemical procedures were carried out in duplicate.

### Antioxidant enzyme activities

Superoxide dismutase (SOD) and catalase (CAT) activities were measured in erythrocytes and plasma, glutathione peroxidase (GPx) and glutathione reductase (GR) activities were determined in erythrocytes, and myeloperoxidase (MPO) activity was determined in plasma by methods previously described [28]. CAT activity was determined by a spectrophotometric method based on the decomposition of H_2_O_2_. SOD activity was determined using a xanthine/xanthine oxidase system to produce the superoxide anion. The produced anion induces the reduction of cytochrome C, which was monitored at 550 nm. GPx activity was determined by an assay that requires H_2_O_2_ and NADPH as substrates and GR as enzyme indicator. The decrease in NADPH absorbance was followed at 340 nm during the oxidation of NADPH to NADP^+^. GR activity determines the rate of conversion of oxidized glutathione (GSSG) to reduced glutathione (GSH) by monitoring the oxidation of NADPH at 340 nm. MPO activity was measured following the oxidation of guaiacol. The reaction mixture contained 13.5 mM guaiacol and the reaction was initiated with 300 μM H_2_O_2_, and the absorbance at 470 nm was monitored. All enzymatic activities were determined at 37°C in a spectrophotometer Shimadzu UV-2100.

### MDA-protein adducts

The levels of plasma MDA-protein adducts were measured by an enzyme immunoassay (Cell Biolabs, Inc.). Briefly, protein samples or standards (10 μg mL^-1^) were adsorbed onto a 96-well plate and incubated overnight at 4°C. MDA–protein adducts were visualized with a specific anti-MDA antibody, followed by an HRP-conjugated secondary antibody. The concentration of MDA-protein adducts in the samples was calculated using a standard curve of known concentrations.

### Nitrite determination

Nitrite levels were determined in plasma by the acidic Griess reaction using a spectrophotometric method. Plasma was deproteinized with acetone and kept overnight at -20°C. Samples were centrifuged at 15,000g for 10 min at 4°C, and supernatants were recovered. A 96-well plate was loaded with the samples or nitrite standard solutions in duplicate. Sulfanilamide in 5% HCl and N-(1-napthyl)-ethylenediamine (0.1% w/v) in water was then added to each well. The absorbance at 540 nm was measured following an incubation of 30 min.

### VEGF and endothelin-1 immunoassay

Quantification of VEGF and endothelin-1 in plasma was performed using a commercially available immunoassay kits (Assay Designs, Inc., Ann Arbor, MI) following the manual instructions. All determinations were realized in duplicate, and absorbance was measured using a microplate reader at 450 nm.

### IL-6 determination

Plasma IL-6 was determined using an ELISA kit (Diaclone, lit for GENPROBE) following the manufacturer’s instructions for use. The overall intra-assay coefficient of variation was calculated to be 4.4% for IL-6; the calculated overall inter-assay coefficient of variation was 9.1% for IL-6.

### Statistical analysis

Statistical analysis was performed using Statistical Package for Social Sciences (SPSS version17.0). Data are expressed as mean ± SEM and considering p < 0.05 statistically significant. To assess the normal distribution of the data the Kolmogorov–Smirnov test was applied. The statistical differences between the obtained data were evaluated by two-way analysis of variance (ANOVA). The analyzed factors were the HBOT (HBOT), the number of the session (T) and their possible interaction (Int). When significant differences were reported, a post hoc testing (DMS) was used to establish the differences between the different data. Student’s t-test for paired data was used for hematological parameters, CK activity and MDA levels before the first session and 1 month after wound recovery.

## Results

Previous conventional treatments to heal the chronic wounds (including antibiotics, topical dressings, and debridement of tissue) were ineffective in these patients. The patients presented wounds with an average time without symptoms of healing of 20.2±10.0 months when they began with the HBOT. All patients participating in the present study were recovered after 20 HBOT sessions. The diabetic wound recovery in the patients’ foot before the first HBOT session was observed, being this recovery higher after the last HBOT session. The statistical analysis addressed to determine if there were differences between the diabetic and non-diabetic patients reported no significant differences in any of the parameters studied (data not shown).

Changes in hematological parameters, serum CK activity and MDA concentration in samples from wounded patients obtained before and after the sessions 1, 5 and 20 of the HBO treatment are shown in Table 1. Significant statistical effects were not evidenced in any on the hematological parameters analyzed. The duration of HBOT significantly influenced serum CK activity with a progressive decrease along the sessions. The results at the session 5 were significantly lower than the ones from the first session and the results from the session 20 were also lower than the ones found in the sessions 1 and 5; finally, at the session 20, serum CK activity attained a 54% reduction with respect to the session 1 (initial value). Similarly, the plasma concentration of MDA adducts was significantly influenced by the treatment duration; plasma MDA was significantly reduced at the last session with respect to the session 1 (p < 0.05). In addition, the hematological parameters did not report any significant differences between the data before the first treatment and 1 month after wound healing. On the contrary, CK activity and MDA adducts were significantly lower 1 month after wound recovery respect to initial values.

**Table 1:** Hematological parameters, creatine kinase activity and MDA in samples obtained from wounded patients (n = 14) before and after the sessions 1, 5 and 20 of the HBO treatment and 1 month after wound recovery.

	Session 1		Session 5		Session 20		1 month after wound healing
	Before	After	Before	After	Before	After	
**Erythrocytes (10**^**6**^**·μL**^**-1**^**)**	4.18 ± 0.08	4.23 ± 0.07	4.11 ± 0.07	4.16 ± 0.06	4.12 ± 0.08	4.17 ± 0.11	4.24 ± 0.11
**Hematocrit (%)**	38.9 ± 0.6	39.0 ± 0.6	38.0 ± 0.5	38.8 ± 0.5	38.4 ± 0.6	39.0 ± 0.8	39.4 ± 0.7
**Hemoglobin (g·dL**^**-1**^**)**	12.9 ± 0.8	13.0 ± 0.9	12.6 ± 0.8	12.8 ± 0.9	12.7 ± 1.0	12.8 ± 1.2	12.9 ± 0.7
**Leukocytes (10**^**3**^**·μL**^**-1**^**)**	5.99 ± 0.16	6.35 ± 0.18	6.38 ± 0.16	6.39 ± 0.17	6.64 ± 0.23	6.61 ± 0.28	5.79 ± 0.28
**Creatine kinase (U/L)**	258 ± 29	231 ± 26	181 ± 15 ^#^	174 ± 15 ^#^	119 ± 9.1 ^$^	124 ± 9.2 ^$^	123 ± 15 *
**MDA (μmol·mL**^**-1**^**)**	0.51 ± 0.02	0.48 ± 0.01	0.44 ± 0.02	0.42 ± 0.03	0.34 ± 0.02 ^#^	0.32 ± 0.02 ^#^	0.31 ± 26 *

The effects of HBOT sessions and before/after data were evaluated by one way ANOVA, P < 0.05,

^#^ indicates significant differences respect to session 1.

^$^ indicates significant differences respect to sessions 1 and 5.

Significant differences between the data before the first treatment and 1 month after wound healing were analysed by Student’s t-test for paired data,

* P < 0.05.

**Fig 1:**
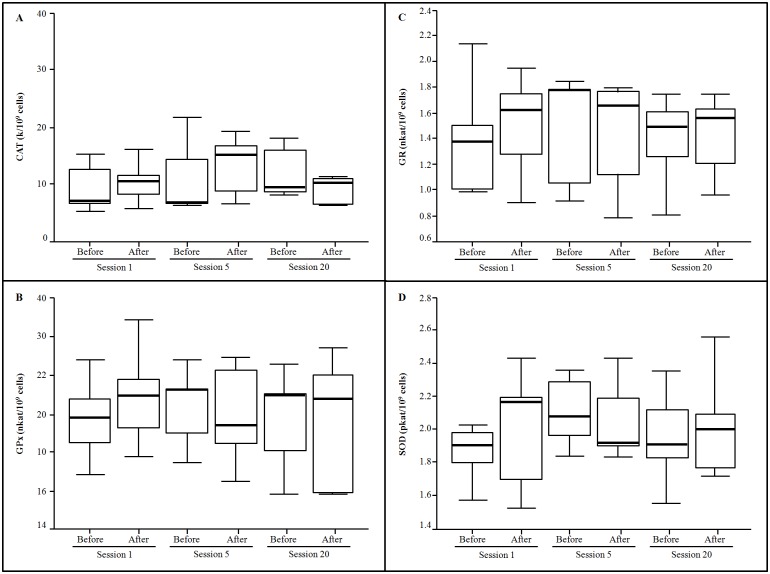
Erythrocyte enzyme activities obtained from wounded patients (n = 14) before and after the sessions 1, 5 and 20 of the HBO treatment. One way ANOVA, P < 0.05, * indicates significant differences between samples obtained before and after HBO treatment.

Erythrocyte enzymatic activities are reported in Fig 1. No significant effects of HBOT and treatment duration on erythrocyte antioxidant activities were observed. The initial antioxidant enzymatic activities of erythrocytes were maintained after each HBOT session and along the treatment.

**Fig 2:**
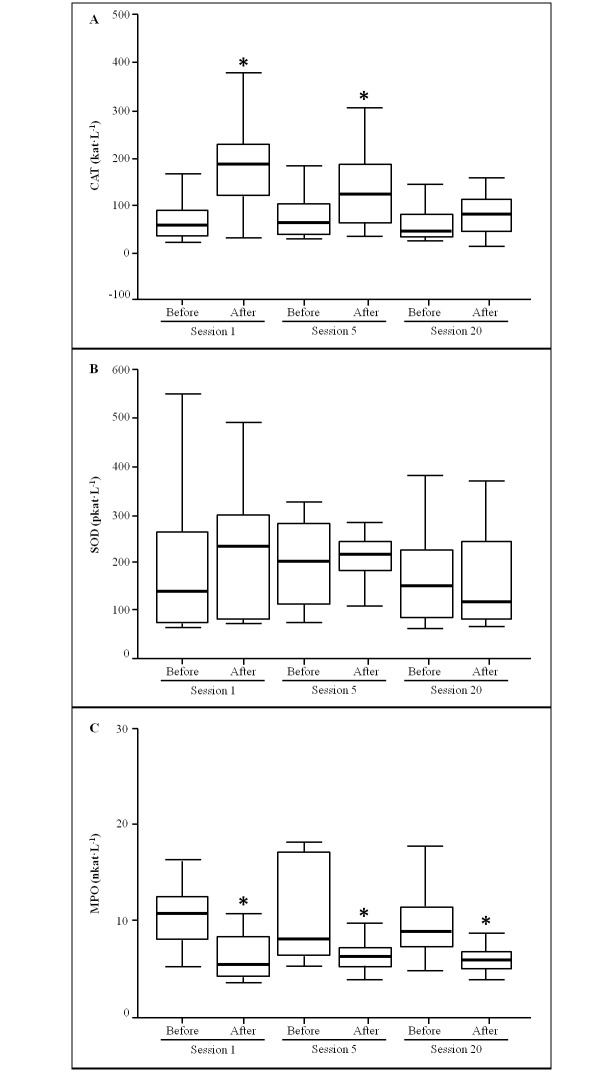
Plasma enzyme activities obtained from wounded patients (n = 14) before and after the sessions 1, 5 and 20 of the HBO treatment. One way ANOVA, P < 0.05, * indicates significant differences between samples obtained before HBO treatment and after HBO treatment.

Plasma enzymatic activities are reported in Fig 2. Plasma SOD activity was not influenced by HBOT and treatment duration with similar values in all situations. Plasma catalase activity responded to the HBOT, but not to the duration of the treatment, with a significant raise after the sessions 1 and 5 (p < 0.05), although this increase was not significant after 20 HBOT sessions. A significant reduction of plasma MPO activity was evidenced after each analyzed HBOT sessions while duration treatment did not affect it.

**Fig 3:**
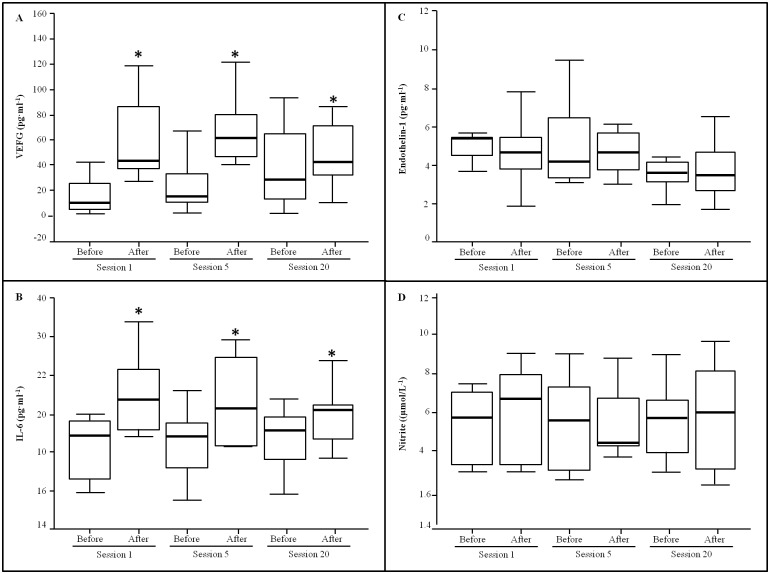
Plasma nitrite, VEGF, endothelin-1 and IL-6 levels. Nitrite, VEGF, endothelin-1 and IL-6 results in plasma obtained from wounded patients (n = 14) before and after the sessions 1, 5 and 20 of the HBO treatment. One way ANOVA, P < 0.05, * indicates significant differences between samples obtained before HBO treatment and after HBO treatment, # indicates significant differences respect to the session 1.

Plasma VEGF, endothelin-1 and IL-6 levels are presented in Fig 3. VEGF and IL-6 levels reported a significant increase after each HBOT session respect to the pre-session values (p < 0.05), without differences between the basal values of session 1, 5 and 20. A progressive decrease in endothelin-1 levels were observed during the HBOT, although statistical differences were only evidenced at the session 20 with respect to the session 1 (p < 0.05). Plasma nitrite levels as a marker of the NO synthesis, a blood pressure modulator remained unchanged during all the HBOT.

## Discussion

The patients included in this study had infections and/or venous or arterial insufficiency with long-standing wounds lasting for mean of 20.2 ± 10 months, without having experienced any improvement. In the present study, the HBOT for 1 month (20 HBOT sessions) has showed to induce considerable decreases in the wound size and fast healing rates similar to previous studies [11]. Patients with chronic wounds have high values of oxidative and tissue damage markers including MDA levels and CK activity respect reference values and the values determined one month after the wound healing [29, 30]. The decrease observed in the oxidative damage markers during HBOT is in accordance with the recovery process reported in the patients. In this way, it has been reported that the subjects with first myocardial infarction which received thrombolytic therapy alone evidenced a higher peak of CK activity respect to a HBOT group [31]. However, some studies have showed small changes in markers of lipid peroxidation in plasma after HBOT [32, 33]. In a prior study performed with healthy subjects, HBOT did not induce alterations in markers of oxidative damage in plasma and lymphocytes, but it induced an antioxidant response characterized by an increased lymphocyte GPx activity and hemoxygenase-1 (HO-1) mRNA levels, which participates in a inflammatory pro-resolving circuit [34]. In addition, no significant effect by HBOT and treatment duration was evidenced in hematological parameters and erythrocyte antioxidant enzymes, in agreement with previous results [32, 35].

The therapeutic basis behind HBOT is the increase in the quantity of dissolved oxygen carried by the blood leading to a significant increase in oxygen concentration in the tissues of the body. High oxygen concentration could increase reactive oxygen species (ROS) production; but paradoxically HBOT induces an antioxidant environment in plasma by increasing the plasma catalase activity. Diverse studies have evidenced increases in the total plasma antioxidant capacity determined after a session with HBOT [36–38]. Moreover, it has also shown that HBOT may operate as a hormetic agent via activating antioxidant and cytoprotective genes in order to protect against a stressful situation [39]. The increase of ROS levels could mediate the expression of key molecules of inflammation, resolution and wound repair. In this way, this increase can be considered the primary mechanism of action for HBOT in wound healing [40]. HBOT could increase ROS generation, inducing a protective and adaptative response to help cells and tissues to manage different endogenous and environmental stressors more efficiently. The increased production of ROS after HBOT activates the nuclear factor κβ (NFκβ) which up-regulates several pathways involved in the initiation of the protective mechanisms [41]. At sites of neovascularization, ROS stimulate growth factor synthesis by enhancing synthesis and stabilizing HIF-1 [40]. Plasma MPO activity significantly decreased after each HBOT session throughout the experimental period. The observed decrease in the plasma MPO activity could be indicative of a reduced activation of neutrophils. It has been reported that hyperbaric oxygen exposure at 3 ATA for 45 min prevents leukocyte adherence mediated by B_2_ integrins, protecting tissues from injury, although it did not reduce neutrophil viability and functions in response to chemoattractants [42, 43]. Moreover, reductions in the blood supply in wounded tissues can alter the mitochondrial electron transport chain increasing electron leakage and consequently increasing ROS production [44, 45]. HBOT induces the ROS production which can act as signals mediating physiologic responses in mitochondria. HBOT preserves mitochondrial integrity via maintenance of mitochondrial membrane potential and reduction of the mitochondrial pathway of apoptosis [46]. In accordance, repetitive sessions of HBOT could induce adaptations to the oxidative machinery of neutrophils in order to decrease the responsiveness against stimulus, diminishing the inflammatory response and improving the recovery process in the damaged tissues. In this sense, HBOT sessions promote an anti-inflammatory response similar to the resolution inflammatory response by lipid mediators including resolvins and several cytokines [3]. Resolvins exert strong anti-inflammatory effects by blunting excessive neutrophil infiltration into tissues and decreasing the production of pro-inflammatory mediators [3, 47]. Diabetic patients have an altered wound healing which is attributed to diabetic impairment of wound healing resolution phase, even it is pointed out that stimulation of the resolution phase with pro-resolving lipid mediators as resolvins could be a new approach to treat chronic, non-healing wounds in diabetic patients [47]. IL-6 is a pleiotropic cytokine with pro-inflammatory and anti-inflammatory functions [48]. In response to an acute infection, trauma or exercise, acute elevations of IL-6 mediate anti-inflammatory effects, whereas chronic elevated levels of IL-6 are associated with pathogenic inflammation and with detrimental effects on metabolism [49]. IL-6 has been reported to play a role in the resolution of inflammation and to achieve a satisfactory wound healing [26]. IL-6 enhances the induction of macrophages by an alternatively pathway, which are characterized by their anti-inflammatory and wound healing capability [26]. HBOT treatment increased IL-6 plasma levels, which in turn could promote alternative activated macrophages in the wounded tissue driving to the resolution phase of healing wounds. The repetitive sessions of HBOT produced repetitive inputs of IL-6 that participated in the wound healing.

VEGF is an important angiogenic factor with a great growth-stimulatory effect on endothelial cells, and also with a potent mediator effects on vascular permeability [50]. HBOT results in a moderate ROS production, which can induce the release of VEGF and consequently can promote angiogenesis [51]. The ROS, produced during wound healing, may participate as signaling molecules that regulate the VEGF production and diverse cellular responses. HBOT induces VEGF expression which in turn stimulates angiogenesis and improves the recovery process. In a study with human umbilical vein endothelial cell model, it was reported a significant induction in the VEGF gene expression after HBOT [52]. However, only few studies are performed in humans to analyze the effects of HBOT on plasma VEGF concentration, and the results found are controversial [53, 54], possibly as a consequence of different HBOT, experimental procedure, and the different type of wounds evaluated. The release of nitric oxide by smooth muscle cells has been indicated to be involved in the signaling cascade that leads to VEGF synthesis and release [55]. In subjects reporting wounds favorably affected by HBOT, an increase in the levels of nitric oxide was observed after HBOT [9]. Nevertheless, although the nitrite levels obtained in the present study tend to increase 2 hours after each HBOT session, no significant changes were reported. It can be suggested that nitric oxide levels early peaked after the HBOT session and consequently, the measured nitrite returned to background levels after 2 hours.

Endothelin-1 is a strongly vasoconstrictive factor produced by vascular endothelial cells playing a central function in the regulation of basal vascular tone [56]. Endothelin-1 evidenced significant lower values after 20 sessions of HBOT than the initial values. These decreased levels could be necessary to maintain an adequate vascular tone after HBOT and to increase the vascular blood flow in order to facilitate the oxygen availability to wounded tissues. Nitric oxide was reported to effectively suppress the release and the physiological action of endothelin-1 and to nitrosylate endothelin receptors reducing the affinity for endothelin-1 [57]. Consequently, and in addition to its direct vasodilator effects, nitric oxide can indirectly induce vasodilation by inhibiting the release of endothelin-1 [58].

The reduced number of cases in our study and the different varieties of wound etiologies could be a limitation; however, since this is a plasma study, the central responses to HBOT may not depend on the wound type. The loss of patients throughout the study is another limitation arising from the occurrence of complications or abandonment of the protocol for its long duration. Controlled studies with larger number of patients are required for further analysis. In conclusion, HBOT may improve the healing process in chronic wounds. HBOT is a potential therapeutic tool to treat chronic non-healing wounds derived from pathological conditions compromising blood supply and tissue oxygen availability. Although a variety of wound types was included in the current study, the healing wound after HBOT was achieved. The present data evidenced that HBOT regulates wound healing by a common mechanism to several pathologies that includes a plasmatic antioxidant response, the induction of an angiogenic response, the regulation of vascular tone, and switching to resolution of inflammation via increasing the release of IL-6. However, more strong investigations with a greater number of patients and HBOT non-treated patients as control group are necessary so as to increase the understanding of the molecular and beneficial effects of HBOT in the treatment of chronic wounds.

## Supporting Information

S1 Tables Tables including the presented data. (PDF)
