# Supplementary material for: Antioxidant Response of Chronic Wounds to Hyperbaric Oxygen Therapy
Source: PLoS One. 2016 Sep 21;11(9):e0163371. doi: 10.1371/journal.pone.0163371 (PMC5031445; doi:10.1371/journal.pone.0163371)
Supplement: S1 Tables — (PDF) [file pone.0163371.s001.pdf]

Supporting Information

Tables including the presented data

Table 1. Hematological parameters, creatine kinase activity and MDA levels.

	**Before treatment**	**1 month after wound healing**
**Erythrocytes ($10^6 \cdot \mu L^{-1}$)**	4.18 ± 0.08	4.24 ± 0.11
**Hematocrit (%)**	38.9 ± 0.6	39.4 ± 0.7
**Hemoglobin ($g \cdot dL^{-1}$)**	12.9 ± 0.8	12.9 ± 0.7
**Leukocytes ($10^3 \cdot \mu L^{-1}$)**	5.99 ± 0.16	5.79 ± 0.28
**CK (U/L)**	258 ± 29	123 ± 15 *
**MDA ($\mu mol/ \cdot mL^{-1}$)**	0.52 ± 0.03	0.31 ± 26 *

Hematological parameters, CK activity and MDA levels in samples obtained from wounded patients (n=14) before the first session and 1 month after wound recovery. Student's t-test for paired data, $P < 0.05$, * indicates significant differences.

Table 2. Hematological parameters and creatine kinase activity

	**Session 1**		**Session 5**		**Session 20**	
	**Before**	**After**	**Before**	**After**	**Before**	**After**
**Erythrocytes ($10^6 \cdot \mu L^{-1}$)**	4.18 ± 0.08	4.23 ± 0.07	4.11 ± 0.07	4.16 ± 0.06	4.12 ± 0.08	4.17 ± 0.11
**Hematocrit (%)**	38.9 ± 0.6	39.0 ± 0.6	38.0 ± 0.5	38.8 ± 0.5	38.4 ± 0.6	39.0 ± 0.8
**Hemoglobin ($g \cdot dL^{-1}$)**	12.9 ± 0.8	13.0 ± 0.9	12.6 ± 0.8	12.8 ± 0.9	12.7 ± 1.0	12.8 ± 1.2
**Leukocytes ($10^3 \cdot \mu L^{-1}$)**	5.99 ± 0.16	6.35 ± 0.18	6.38 ± 0.16	6.39 ± 0.17	6.64 ± 0.23	6.61 ± 0.28
**CK (U/L)**	258 ± 29	231 ± 26	181 ± 15 $^{\#}$	174 ± 15 $^{\#}$	119 ± 9.1 $^{\$}$	124 ± 9.2 $^{\$}$

Hematological parameters and CK activity in samples obtained from wounded patients (n=14) before and after the sessions 1, 5 and 20 of the HBO treatment. One way ANOVA, $P < 0.05$, # indicates significant differences respect to session 1. $ indicates significant differences respect to sessions 1 and 5.

Table 3. Erythrocyte and plasma enzyme activities

	Session 1		Session 5		Session 20	
	**Before**	**After**	**Before**	**After**	**Before**	**After**
**Erythrocytes**						
**CAT ($k/10^9$ cells)**	9.34 ± 0.77	9.89 ± 0.52	10.9 ± 1.5	13.4 ± 1.4	11.9 ± 1.1	12.0 ± 1.7
**GPx ($nkat/10^9$ cells)**	19.6 ± 0.4	20.7 ± 0.5	20.5 ± 0.5	19.9 ± 0.6	19.7 ± 0.6	20.0 ± 0.6
**GR ($nkat/10^9$ cells)**	1.39 ± 0.07	1.51 ± 0.07	1.47 ± 0.09	1.44 ± 0.10	1.38 ± 0.08	1.41 ± 0.06
**SOD ($pkat/10^9$cells)**	1.92 ± 0.04	2.02 ± 0.06	2.09 ± 0.04	2.03 ± 0.05	1.96 ± 0.06	1.98 ± 0.05
**Plasma**						
**CAT ($kat·L^{-1}$)**	72.4 ± 9.8	177 ± 35*	73.5 ± 8.1	141 ± 31*	72.8 ± 12.2	85.7 ± 7.6
**SOD ($pkat·L^{-1}$)**	202 ± 33	218 ± 27	197 ± 20	210 ± 14	172 ± 20	179 ± 21
**MPO ($nKat·L^{-1}$)**	10.8 ± 0.9	6.16 ± 0.51*	10.7 ± 1.1	6.32 ± 0.30*	9.47 ± 0.72	7. 23 ± 0.88*

Erythrocyte and plasma enzyme activities obtained from wounded patients (n=14) before and after the sessions 1, 5 and 20 of the HBO treatment. One way ANOVA, $P < 0.05$, * indicates significant differences between samples obtained before HBO treatment and after HBO treatment.

Table 4. Plasma nitrite, VEGF, endothelin-1 and IL-6 levels

	Session 1		Session 5		Session 20	
	**Before**	**After**	**Before**	**After**	**Before**	**After**
**Nitrite (μmol/L^{-1})**	5.40 ± 0.61	5.95 ± 0.82	5.32 ± 0.79	5.84 ± 0.85	5.37 ± 0.64	5.68 ± 0.91
**VEGF (pg·ml^{-1})**	16.3 ± 3.4	59.3 ± 8.1 *	24.3 ± 7.4	69.2 ± 7.2 *	36.0 ± 7.7	48.4 ± 9.6 *
**Endothelin-1 (pg·ml^{-1})**	5.02 ± 0.18	4.71 ± 0.41	5.08 ± 0.56	4.72 ± 0.28	3.55 ± 0.20 #	3.77 ± 0.39 #
**IL-6 (pg·ml^{-1})**	35.2 ± 3.4	51.2 ± 4.1 *	36.4 ± 3.9	49.1 ± 5.2 *	37.7 ± 3.4	45.4 ± 3.3 *

Nitrite, VEGF, endothelin-1 and IL-6 results in plasma obtained from wounded patients (n=14) before and after the sessions 1, 5 and 20 of the HBO treatment. One way ANOVA, $P < 0.05$, * indicates significant differences between samples obtained before HBO treatment and after HBO treatment, # indicates significant differences respect to the session 1.
